# Sustained effects of faculty leadership development modules for clinical instructors of core competences education in Taiwan: a four-year explanatory case study

**DOI:** 10.1186/s12909-020-02065-w

**Published:** 2020-05-15

**Authors:** Fa-Yauh Lee, Ying-Ying Yang, Chia-Chang Huang, Ling-Ju Huang, Ching-Chih Chang, Jen-Feng Liang, Shiau-Shian Huang, Wei-Shin Lee, Dai-Yin Lu, Chiao-Lin Chuang, Ling-Yu Yang, Hui-Chun Huang, Boaz Shulruf, Chen-Huan Chen, Shou-Yen Kao

**Affiliations:** 1 Division of General Medicine, Taipei Veteran General Hospital, Taipei, Taiwan; 2 Department of Medicine, Taipei Veterans General Hospital, Taipei, Taiwan; 3 National Yang-Ming University, Taipei, Taiwan; 4 Division of Clinical Skills Training Center, Taipei Veteran General Hospital , Taipei, Taiwan; 5 Faculty of medicine, School of Medicine, National Yang-Ming University, Taipei, Taiwan; 6 Department of Medicine, Taipei Veteran General Hospital , Taipei, Taiwan; 7 Department of Medical Education, Taipei Veteran General Hospital , Taipei, Taiwan; 8 Bali Psychiatric Center, Ministry of Health and Welfare, Bali, Taiwan; 9 New South Wales Sydney University, Sydney, Australia

**Keywords:** Clinical instructor, Core competence education, Leadership, Sustainability

## Abstract

**Background:**

The Accreditation Council for Graduate Medical Education (ACGME) core competencies (CC) in general medicine-based primary care are essential for junior medical trainees. In this country, a *regular* faculty development (FD) program aimed at training faculty in instructing (teaching and assessing) these CC had operated. However, leadership was not emphasized. In a new *intervention* module, the roles and associated responsibilities of *clinical instructors* to *conduct*, *design*, and *lead* CC-based education were emphasis.

**Aims:**

This follow-up explanatory case study compares the effectiveness of *intervention* module with that of the previous *regular* module.

**Methods:**

The *regular* group (*n* = 28) comprised clinical instructors who participated in the FD module during the 2013–2014 year while the *intervention* group (*n* = 28) was composed of 2015–2016 participants. Prior to the formal (*hands-on*) training, participants in the *intervention* group were asked to study the online materials of the *regular* module. These participants then received a 30-h hands-on training in *conducting*, *designing*, and *leading* skills. Finally, they prepared a 10-h reflective end-of-module presentation of their real-world practices.

**Results:**

Following the training, a higher degree improvement in participants self-reported familiarity with CC education, self-confidence in their ability to deliver CC education and sustained involve CC education were noted among the *intervention* FD group, compared with the *regular* FD group. In the *intervention* group, *senior* academicians (associate and full professor) are more substantially involved in *designing* and *leading* CC-based courses than junior academicians (lecturers and assistant professors). Among non-teaching award winners of in the *intervention* FD group, the follow-up degree of sustained involvement in *delivering, designing and leading* CC-based courses was significantly higher than that of the *regular* group.

**Conclusions:**

Our study demonstrated that leadership training in the *intervention* FD modules substantially motivated clinical instructors to become leaders in CC education.

## Background

The Accreditation Council for Graduate Medical Education (ACGME) core competencies (CC), in general medicine-based primary care are essential for junior medical trainees. They included medical knowledge, interpersonal and communication skills, system-based practice, practice-based learning and improvement, professionalism and patient care. Clinical instructors must teach and assess junior medial trainees in CC before they enter sub-specialties. In 2003, the outbreak of the severe acute respiratory syndrome (SARS) in this country exposed serious deficiencies of CC-based primary care by junior trainees due to a lack of appropriate education. Accordingly, there is an urgent educational need to train faculty to educate junior medical trainees in CC-based primary care. Faculty development (FD) refers to activities that clinical instructors or organization pursue to improve their knowledge, skills, and behaviors in response to specific educational needs [1, 2].

In 2003, during the post-SARS era, Executive Yuan of the Department of Health (DOH), announced a nationwide pilot FD program to cultivate clinical instructors whose are familiar with, have confidence in, and are substantially involved in CC-based education (teachings and assessments) [1–5]. In 2009, the DOH began to regularly fund “FD programs” in nationwide organizations to strengthen clinical instructors’ skills for *delivering* CC education [5]. The *regular* FD model supported by situated and experiential learning theories that emphasized on-site observational learning and guided reflection had been continuously utilized in our hospital [6, 7]. In general, clinical instructors had reported that our *regular* FD module familiarizes them with CC-based teaching and assessment and thus, increases their confidence [8, 9].

Moreover, since 2013, clinical instructors in this country have been facing new challenges, including the extension of postgraduation training from 3 months to 2 years, an amendment in the number of years of medical school study from seven to six, and limitation on work hours for all residents [10–12]. Accordingly, clinical instructors are expected to competently show the way in *delivering, designing* and *leading* CC education that fits the need of a system in the midst of reform [13–15].

However, a survey of participants revealed that our previous *regular* FD module did not effectively cultivate their ability to play multiple roles (instructors and leaders) or to take on corresponding responsibilities (*delivering*, *designing* and *leading* CC education) [14, 15]. The aims of implementing leadership in FD is to train leaders who can solve challenges in medical practice and education [14, 15]. In addition to train in *delivering* skills as in the *regular* FD module, the *intervention* module emphasized the training of *designing* and *leadership* skills [16, 17]. After participating in the *intervention* FD module, clinical instructors are expected to competently show the way in CC education *deliver*, *design*, and *leadership*,

This study aimed to compare the effects of the *intervention* and *regular* FD modules on participants’ familiarity with CC education, confidence in their delivery, and sustained involve in instruction and leadership roles in CC education following training. Moreover, the various impacts of this *intervention* module on participants with different academic positions and teaching performance were compared.

## Methods

### Study design

An explanatory case study is an in-depth exploration and explanation of an intervention in a real-life context, opposing to hypothesis testing [18, 19]. This research involved an explanatory case study that evaluated how and why the new intervention worked [20]. Age and sex-matching of new clinical instructors were voluntarily included for comparison between *regular* and *intervention* groups.,

### Setting

#### Previous *regular* FD module for training clinical instructors

With respect to CC-based teaching and assessment delivery skills, the major training topics focused on principles of identifying learning objectives, adult learning, creating and maintaining a positive learning environment, developing and using interactive audiovisual tools, and on-site observational learning and guided reflection.

**Fig. 1:**
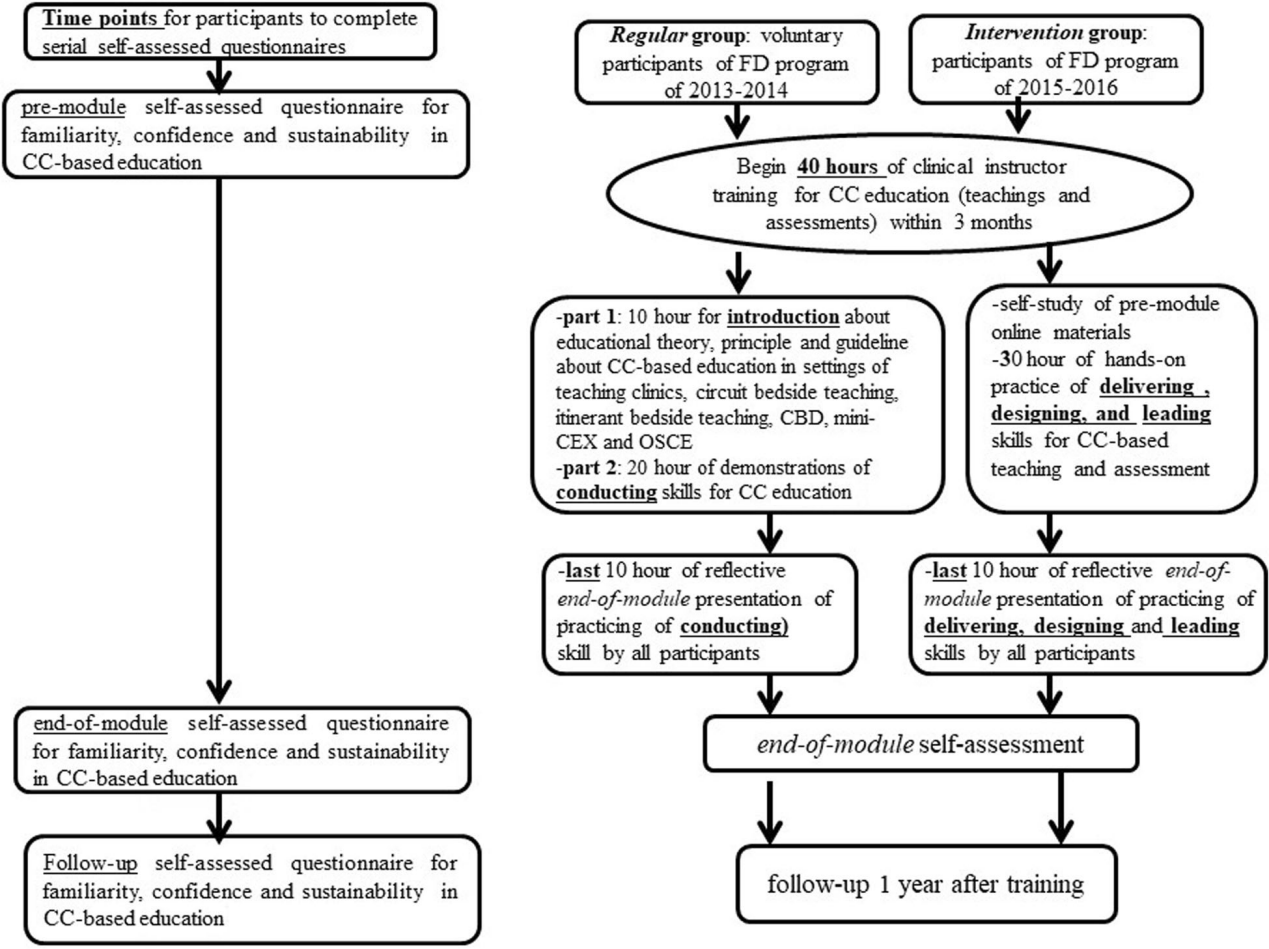
Schematic Diagram of the entire study: CC: core competence, CBD: case-based discussion, mini-CEX: minimal clinical examination exercise, OSCE: objective structural clinical examination

As our previous report indicated [8, 21], the *regular* FD module comprised 40 h across 3 months. It consisted of brief expository lectures and small group discussions. The first 30 h included an introduction to educational theory, and on-site observation of CC education *delivering* skills. On the final 10 h, as part of the *end-of-module* presentation, presenter reflected their skills *in delivering* CC education and received paper and face-to-face feedback from senior facilitators and peers (Fig. 1 and Table 1), following an interactive discussion.

**Table 1:** comparison between *regular* and i*ntervention* faculty development (FD) modules

	***Regular*** FD module	***Intervention*** FD module
**Teaching strategies**	brief expository lectures, on-site observational learning and small group discussions	Pre-module online SDL, on-site observational learning and small group *hands-on* practicing
**Pre-module preparation**	*No need for preparation, knowledges and skills for CC-based education are introduced and discussed in formal lectures	Materials of 30 h of *delivering* skill-focused materials were studied online by participants of *regular* module before class
**Assignments [*End-of-module*presentation]**	each new clinical instructor present within 15 min. and will receive 5 min. Feedback from peer and facilitator	
	Guided reflection of real world practicing of *delivering* skills for CC education and future plan	Guided reflection of real world practicing of *delivering*, *designing* and *leading* skills for CC education and future plan
**Used discussion questions for guided self-reflection at the end of module**	***I am familiar with …*** . *-delivering, designing,* and l*eading* CC-based teachings in teaching clinics, itinerant bedside, circuit bedside as well as CC-based assessments in CBD, mini-CEX and OSCE ***I feel confident about … .****.* - *delivering, designing,* and l*eading* CC-based teachings in teaching clinics, itinerant bedside, circuit bedside as well as CC-based assessments in CBD, mini-CEX and OSCE **I*am sustainably … .within 1 year*** -involve in *delivering*, *designing* and *leading* CC-based courses as well as incorporate CC in *delivering*, *designing* and *leading* courses	

*SDL* self-directed learning, CC (Core competencies) indicated the six core ACGME competencies including medical knowledge (MK), interpersonal and communication skills (ICS), system-based practice (SBP), practice-based learning and improvement (PBLI), professionalism (P), Patient care (PC)

After participating in the *regular* FD module, clinical instructors are expected to be competent in the delivery of CC-based *teaching clinics*, circuit and itinerant *bedside teaching* as well as case-based discussion (CBD), mini-clinical evaluation exercise (mini-CEX) and the objective structured clinical examination (OSCE) [8, 21]. All topics in the course were video recorded and edited by teaching assistants for self-directed learning.

#### Background to the development of the new leadership-enhanced *intervention* FD module

With the reform of the education system, the 2013 and 2014 surveys revealed that trained clinical instructors’ familiarity with the skills of *designing* and *leading* CC-based education and their confidence in delivering it was insufficient. Therefore, during the 2015–2016 year, the educational committee organized a new *intervention* FD module emphasizing leadership. Case-control studies sometimes use historical controls, if controls are not permitted, based on special conditions such as the learning right of participants and educational ethics [22, 23]. Therefore,, historical *regular* FD cohorts were used in this study between 2013 and 2016 as controls for the *intervention* FD cohorts. Notably, both the *regular* and *intervention* FD cohorts (*n* = 81) had similar characteristics (Table 2) and were taught by some senior faculty teams. Of 81 invited clinical instructors, 66 (81.4%) agreed to participate in this study.

**Table 2:** Basal characteristics of participants of *regular* and *intervention* FD modules (*n* = 28 in each group)

	*regular* FD module participants	*intervention* FD module participants
Age, years, mean (SD)	43.8 (5.9)	46.3(8.6)
Male, (%)	67%	64%
Junior academician (lecturer/assistant professor, overall, %)	34/33/67%	36/27/63%
Senior academician (associate/full professor, overall, %)	26/7/33%	30/6/37%
Teaching-award winner within 3 years before training (%)	29%	33%
Specialty of new trained instructors (%)		
Internal medicine/Surgery/Gynecology/Pediatrics/Emergency medicine/ others (Neurology, Psychiatrics, Rehabilitation, Family Medicine, etc) (%)	36/13/13/15/12/11%	32/10/13/15/16/14%
Prior participation in training of clinical teaching or assessment	52%	48%
Junior attending physician (%)	64%	70%
Senior attending physician (%)	35%	30%

Annual teaching-award for teaching performance of teachers are online selected by learners to receive the award; junior or senior attending physician indicated teacher with less than or more than 15 years of being as attending physician

#### *Intervention* FD module for training of clinical instructors in the leadership

In the *intervention* FD module, prior to *hands-on* sessions, participants were asked to study the online materials of the *regular* module [8, 21]. With respect to training leadership, the first 30 h of the *intervention* module focused on *delivering*, *designing* and *leading* CC-based teachings and assessments. For example, participants received hands-on experience with *delivering*, *designing* and *leading* OSCE throught the assessment of six aspects of CCs at different stations with well-designed scenarios and checklists. Different from the *delivery* skills-focused *regular* module, the *end-of-module* presentation during the last the 10 h of *intervention* module highlighted reflection on *delivering*, *designing* and *leading* skills.

### Sustained effects of the three-month training course

After the training, follow-up surveys (at 1 year) were conducted and compared between groups to evaluate whether the newly trained clinical instructors were substantially involved in *delivering*, *designing* and *leading* CC-based education.

### Study outcomes

At the *pre-module*, *end-of-module* and *follow-up* stages of this study, the *intervention* FD module was evaluated and compared with the *regular* FD module. Using a five-point Likert-type self-assessed questionnaire, the degree of familiarity with, self-confidence and sustained involvement in *delivering*, *designing* and *leading* CC-based education was evaluated (Tables 2, 3 and 4, Fig. 1). The questionnaire was based on levels 2 (participants’ familiarity with and confidence their own delivery of in CC-based education) and 3 (participants’ sustained involvement in CC-based education) of Kirkpatrick’s framework to evaluate the effectiveness of the two FD modules. Meanwhile, the participants were encouraged to provide descriptive feedback and discuss with the program director freely. In the subgroup analysis, the impact of academic position and teaching performance (teaching award winner or non-teaching award winner) on the degree of sustained involvements in CC-based teachings and assessments were analyzed the designed in the context of.

**Table 3:** serial evaluation of participants’ familiarity with core competency (CC) teachings and assessments (*n* = 28 in each group)

Questions	*regular* FD module participants			*intervention* FD module participants		
I am familiar with … .. 1.delivering CC-based teachings	pre-module	end-of-module (Δ% from pre-module)	follow-up (Δ% from pre-module)	pre-module	end-of-module (Δ% from pre-module)	follow-up (Δ% from pre-module)
-Teaching clinics	3.5 ± 0.4	4.2 ± 0.3 (20%)	4.3 ± 0.1 (23%)	3.4 ± 0.8	4.6 ± 0.9 (35%)* #1.33	4.7 ± 0.2 (38%)* #4
-Itinerant bedside	3.6 ± 0.2	4.3 ± 0.5 (19%)	4.6 ± 0.7 (28%)	3.7 ± 0.3	4.3 ± 0.5 (16%)	4.5 ± 0.6 (22%)
-Circuit bedside	3.7 ± 0.4	4.6 ± 0.5 (24%)	4.5 ± 0.6 (22%)	3.8 ± 0.2	4.2 ± 0.7 (11%)	4.6 ± 0.3 (21%)
**2. delivering** CC-based assessments						
- Case-based discussion (CBD)	2.9 ± 0.5	3.5 ± 0.3 (21%)	3.7 ± 0.4 (28%)	2.8 ± 0.3	4.2 ± 0.5 (50%)* #2.33	4.4 ± 0.6 (57%)* #1.75
-mini-CEX	3.3 ± 0.2	3.7 ± 0.6 (12%)	3.8 ± 0.3 (15%)	3.1 ± 0.4	4.4 ± 0.6 (42%)* #1.17	4.5 ± 0.2 (45%)* #2.33
-OSCE	2.7 ± 0.6	3.3 ± 0.2 (22%)	3.2 ± 0.4 (19%)	2.9 ± 0.2	4.3 ± 0.3 (48%)* #5	4.2 ± 0.5 (45%)* #2.5
3.**designing** CC-based teachings	1.9 ± 0.4	2.8 ± 0.2 (47%)	3.0 ± 0.4 (57%)	2.1 ± 0.2	4.2 ± 0.5 (100%)** #7	4.4 ± 0.6 (109%)** #3.5
4.**designing** CC-based assessments	2.2 ± .0.8	2.5 ± 0.3 (14%)	2.5 ± 0.7 (14%)	2.3 ± 0.5	3.9 ± 0.3 (70%)* #4.67	4.1 ± 0.4 (78%)* #2.29
5.**leading** CC-based teachings	1.9 ± 0.6	2.2 ± 0.3 (16%)	2.7 ± 0.2 (42%)	2.1 ± 0.3	3.5 ± 0.6 (67%)* #4.33	3.9 ± 0.2 (86%)* #6
6.**leading** CC-based assessments	2.1 ± 0.3	2.3 ± 0.4 (10%)	2.2 ± 0.5 (5%)	1.9 ± 0.8	3.8 ± 0.9 (100%)** #3.75	3.9 ± 0.3 (105%)** #3.4

Data were expressed as mean ± SD; agreement to questions are rated by 5-point Likert scale; 5 = very agree;3 = neutral; 1 = very not agree; *mini-CEX* mini-clinical evaluation exercise; *OSCE* objective structural clinical examination; *, *p* < 0.05 vs. corresponding data of *regular* FD group that analyzed using student *t* tests; Comparison among data of multiple time points were analyzed with ANOVA test; # *t*-test’s effect size for compared data between groups that with significance on *t* test

**Table 4:** serial evaluation of participants’ confidence about core competency (CC) education (*n* = 28 in each group)

Questions	*regular* FD module participants			*intervention* FD module participants		
I feel confident about … …	pre- module	end-of-module (Δ% from pre-module)	Follow-up (Δ% from pre-module)	pre- module	end-of-module (Δ% from pre-module)	Follow-up (Δ% from pre-module)
1. **delivering** CC-based teachings	3.1 ± 0.5	3.9 ± 0.4 (26%)	3.8 ± 0.3 (23%)	3.4 ± 0.2	4.8 ± 0.3 (41%)* #2.3	4.7 ± 0.5 (38%)* #3
2. **delivering** CC-based assessments	2.9 ± 0.2	3.3 ± 0.7 (14%)	3 ± 0.6 (3%)	3.1 ± 0.4	3.7 ± 0.2 (19%)	3.9 ± 0.5 (26%)* #1.5
3.**designing** CC-based teachings	2.2 ± 0.6	2.8 ± 0.4 (27%)	2.7 ± 0.3 (23%)	2.7 ± 0.8	3.4 ± 0.3 (26%)	3.8 ± 0.2 (41%)* #3.67
4.**designing** CC-based assessments	2.3 ± 0.4	2.9 ± 0.5 (26%)	3.1 ± 0.2 (35%)	2.8 ± 0.6	3.6 ± 0.3 (29%)	4.1 ± 0.4 (46%)* #2.4
5**.leading** CC-based teachings	2.4 ± 0.5	3.1 ± 0.2 (29%)	3.4 ± 0.6 (42%)	2.5 ± 0.7	4.0 ± 0.5 (60%)* #4.5	4.1 ± 0.3 (64%)* #1.17
6 **leading** CC-based assessments	1.9 ± 0.3	2.6 ± 0.4 (37%)	2.4 ± 0.2 (26%)	2.3 ± 0.5	3.8 ± 0.5 (65%)* #3	3.9 ± 0.2 (70%)* #7.5

Data were expressed as mean ± SD; agreement to questions are rated by 5-point Likert scale; 5 = very agree; 3 = neutral; 1 = very not agree; *, *p* < 0.05 vs. corresponding data of *regular* FD group that analyzed using student *t* test; Comparison among data of multiple time points were analyzed with ANOVA test; # *t*-test’s effect size for compared data between groups that with significance on *t* test

### Statistics

The *end-of-module* and *follow-up* degrees of familiarity, confidence and sustained involvement in CC-based teachings and assessments between the *regular* and *intervention* groups were analyzed using student *t* tests. The effects of academic position and teaching performance (teaching-award winners or non-teaching-award winners) on the degree of sustained involvements in CC-based education in the *regular* and *intervention* groups were also analyzed using student *t* tests. Additionally, ANOVA was used for the comparison of data among multiple time points. This study was approved by the Ethics Committee of Taipei Veteran General Hospital with ID numbers 2014–02-007 AC and 2015–12-015 BC and performed in compliance with the Declaration of Helsinki [24]. In agreement with these standards, written informed consent was obtained from each participant.

## Results

### Participant characteristics

Of the 66 enrolled clinical instructors, six of them (three *regular* and three *intervention* module participants) were not included in the study because they did not complete all the training. An additional four clinical instructors did not complete all the surveys; yielding a final sample of 56 subjects (*n* = 28 in each group) for final analysis.

Table 2 shows there were no difference in average age, gender distribution, academic level distribution, percentage of teaching award winners, percentage of senior academician (associate and full professor), percentage of distribution of participants from different specialties, percentage of teachers with prior training in clinical teaching or assessment, or percentage of senior physicians (> 15 years as attending physician) between *regular* and *intervention* FD module participants.

### The *intervention* FD module increased clinical instructors’ familiarity with the skills of *delivering*, *designing* and *leading* CC-based education

At the *pre-module* stage, there was no difference in baseline familiarity with the skills of *delivering*, *designing* and *leading* CC-based teachings (teaching clinics and, itinerant, or circuit bedside skills) and assessments (CBD, mini-CEX, or OSCE) between the *regular* and *intervention* groups (Table 3). However, at the end of the module, the *intervention* group exhibited higher degree of familiarity with the above-mentioned skills than the *regular* group. The follow-up data in Table 3 reveal that the participants’ familiarity was sustained for one 1 year.

### The *Intervention* FD module increased clinical instructor’s confidence in the skills of *delivering*, *designing* and *leading* CC-based education

At the *pre-module* stage, there was no difference in baseline confidence concerning *delivering*, *designing* or *leading* CC-based teachings and assessment between the *regular* and *intervention* FD module participants (Table 4). However, at the *end-of-module and follow-up* stages, the level of confidence exhibited by the *intervention* group in the above-mentioned skills was higher than that of the *regular* group.

In the *intervention* group, the participant’s baseline confidence was already high with respect to *delivery* skills. Following the training, at the e*nd-of-module* and *follow-up* stages, the *intervention* group’s CC-based *design* and *leadership* skills were effectively enhanced (Table 4). In other words, the *intervention* FD module effectively trained new clinical instructors as *leaders* in CC-based educations.

### The *Intervention* FD module encouraged new clinical instructors to sustain their involvement in *delivering*, *designing* and *leading* CC-based teaching and assessment

In Table 5, at the pre-module and end-of-module stages, there was no difference in the degree of *delivering* CC-based courses between the *regular* and *intervention* groups. Nevertheless, at the follow-up stage, the *intervention* group exhibited a high degree of sustained involvement in *designing* and *leading* CC-based courses than the *regular* group. These results show that the *intervention* FD module stimulated the participants’ desire to practice complex skills such as *designing* and *leading* CC-based teachings and assessments after the training. Table 6 reveals that the powers (60.7–100%) of the significant parameters at *end-of-module* and *follow-up* stages were acceptable with sample size of 28 in both the *regular* and *intervention* groups and a significance level of 0.05 (α, type I error).

**Table 5:** the sustainability of new trained clinical instructors as leaders of core competence (CC) education

Questions	*regular* FD module’ participants			*intervention* FD module’ participants		
I am sustainably … … .. within 1 year	pre-module	end-of-module (Δ% from pre-module)	Follow-up (Δ% from pre-module)	pre-module	end-of-module (Δ% from pre-module)	Follow-up (Δ% from pre-module)
1. involve in **delivering** CC-based education	3.1 ± 0.2	3.8 ± 0.2 (23%)	3.9 ± 0.3 (26%)	2.8 ± 0.1	3.8 ± 0.2 (36%)	4.0 ± 0.9 (43%)
2.incorporate CC in **delivering** courses	3.3 ± 0.1	3.6 ± 0.1 (9%)	3.7 ± 0.7 (12%)	3.1 ± 0.2	3.6 ± 0.1 (16%)	3.9 ± 0.4 (26%)
3.involve in **designing** CC-based education	2.4 ± 0.3	2.9 ± 0.2 (21%)	3.0 ± 0.3 (25%)	3.2 ± 0.2	4.1 ± 0.2 (28%)	4.3 ± 0.2 (34%)* #4.33
4. incorporate CC in **designing** courses	2.4 ± 0.2	3.1 ± 0.1 (29%)	3.2 ± 0.4 (32%)	2.9 ± 0.1	4 ± 0.3 (38%)	4.1 ± 0.3 (41%)
5. involve in **leading** CC-based education	2.3 ± 0.1	3 ± 0.2 (30%)	3.1 ± 0.6 (35%)	2.7 ± 0.2	4.3 ± 0.2 (59%)	4.3 ± 0.4 (59%)* #2
6.incorporate CC in **leading** courses	2.5 ± 0.2	3.2 ± 0.3 (28%)	3.3 ± 0.4 (32%)	2.6 ± 0.3	4.2 ± 0.2 (62%)	4.4 ± 0.5(69%)* #2.75

Data were expressed as mean ± SD; the degree of agreement to the listed items are rated by 5-point Likert scale; 5 = very agree; 4 = agree;3 = neutral;2 = not agree;5 = not very agree. *, ***p*** < 0.05 vs. corresponding data of participants of *regular* FD module that analyzed using student *t* tests; Comparison among data of multiple timepoints were analyzed with ANOVA test; # *t*-test’s effect size for compared data between groups that with significance on *t* test

**Table 6:** The power analysis of significant parameters in Tables 3, 4 and 5 between *regular* and *intervention* FD modules by self-reported participants’ familiarity, confidence and sustainability for core competency (CC) education after training

Significant parameters	Data of *end-of-module* stage	Data of *follow-up* stage
I am **familiar** with **delivering** CC-based teaching- *Teaching clinics*	60.7%	100%
I am **familiar** with **delivering** CC-based assessment- Case-based discussion (CBD)	100%	99.9%
I am **familiar** with **delivering** CC-based assessment-mini-CEX	99.2%	99.9%
I am **familiar** with **delivering** CC-based assessment- OSCE	100%	100%
I am **familiar** with **designing** CC-based teaching and assessments	100%	100%
I am **familiar** with **leading** CC-based teaching and assessments	100%	100%
I feel **confident** in **delivering** CC-based teachings	100%	100%
I feel **confident** in **leading** CC-based teachings and assessments	100%	100%
I am sustainably **involve** in **designing** and **leading** CC-based education within 1 year	100%	100%
I am sustainably **incorporate CC** in **leading** courses within 1 year	100%	100%

### Senior academicians and non-teaching award winners in the *intervention* group were more sustainably involved in *designing* and *leading* CC education

**Fig. 2:**
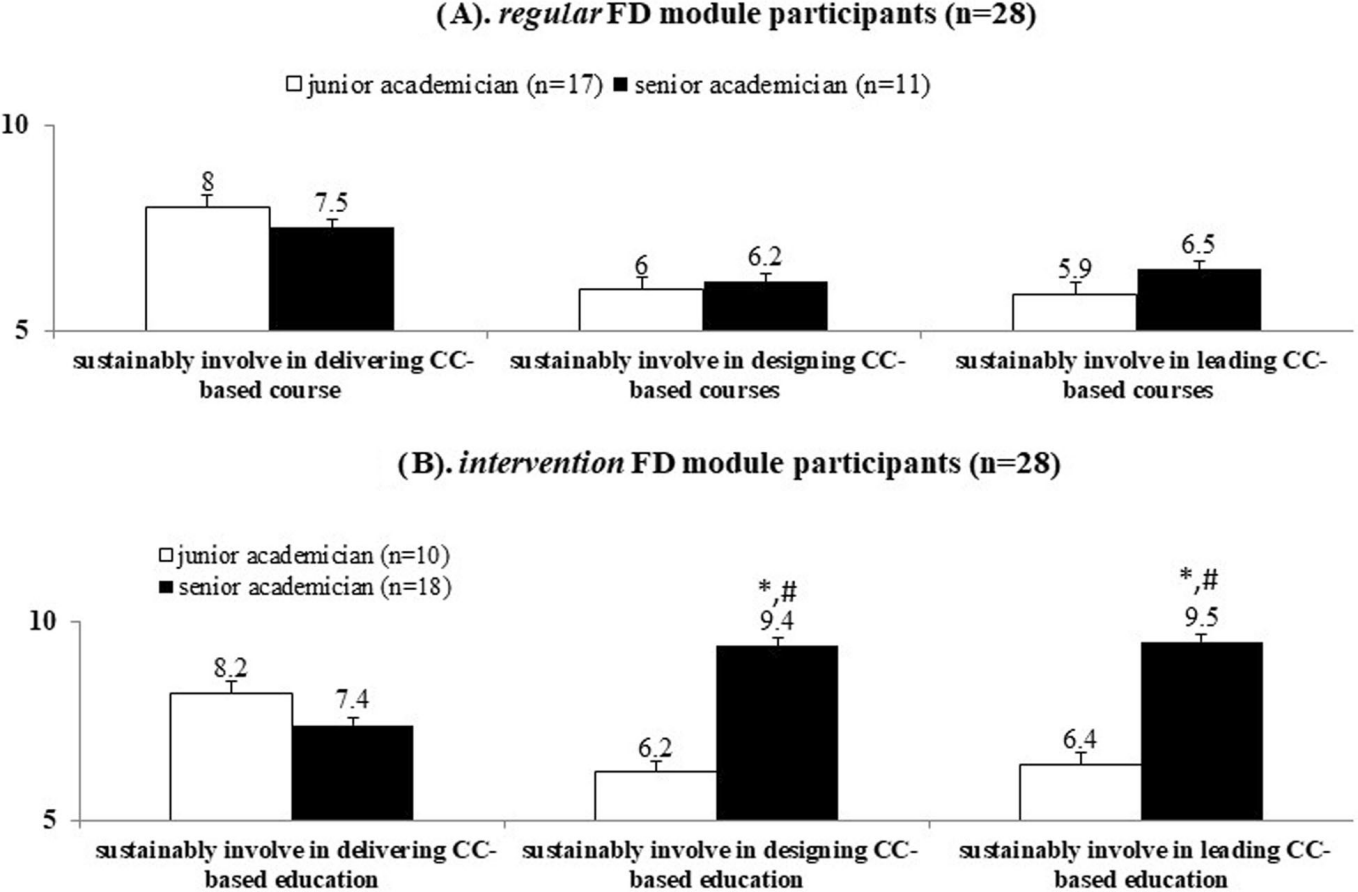
Comparison of the follow-up degree of sustained involvement in core competency (CC)-based teachings and assessments between junior and senior academicians participating in either (**a**) *regular* or (**b**) *intervention* FD modules; *P < 0.05 vs. data of junior academicians; ^#^*P* < 0.05 vs. data compared to the data of the senior academicians in the *regular* group were analyzed

Generally, program directors have more opportunities to *design* and *lead* CC-based teachings or assessments. In fact, most program directors at our institution are senior academicians. In the *regular* group, there was no difference in the follow-up degree of sustained involvement in *delivering*, *designing* and *leading* CC-based teachings or assessments between senior (*n* = 11) and junior (*n* = 17) academicians (Fig. 2a). In other words, the *regular* training did not further increase the senior academicians’ degree of sustained involvement in *designing* and *leading* CC-based teachings or assessments as expected. By contrast, in the *intervention* group, *senior* academicians (*n* = 18) had more sustained involvement in *designing* and *leading* CC-based teachings or assessments than junior academicians (*n* = 10) (Fig. 2b).

**Fig. 3:**
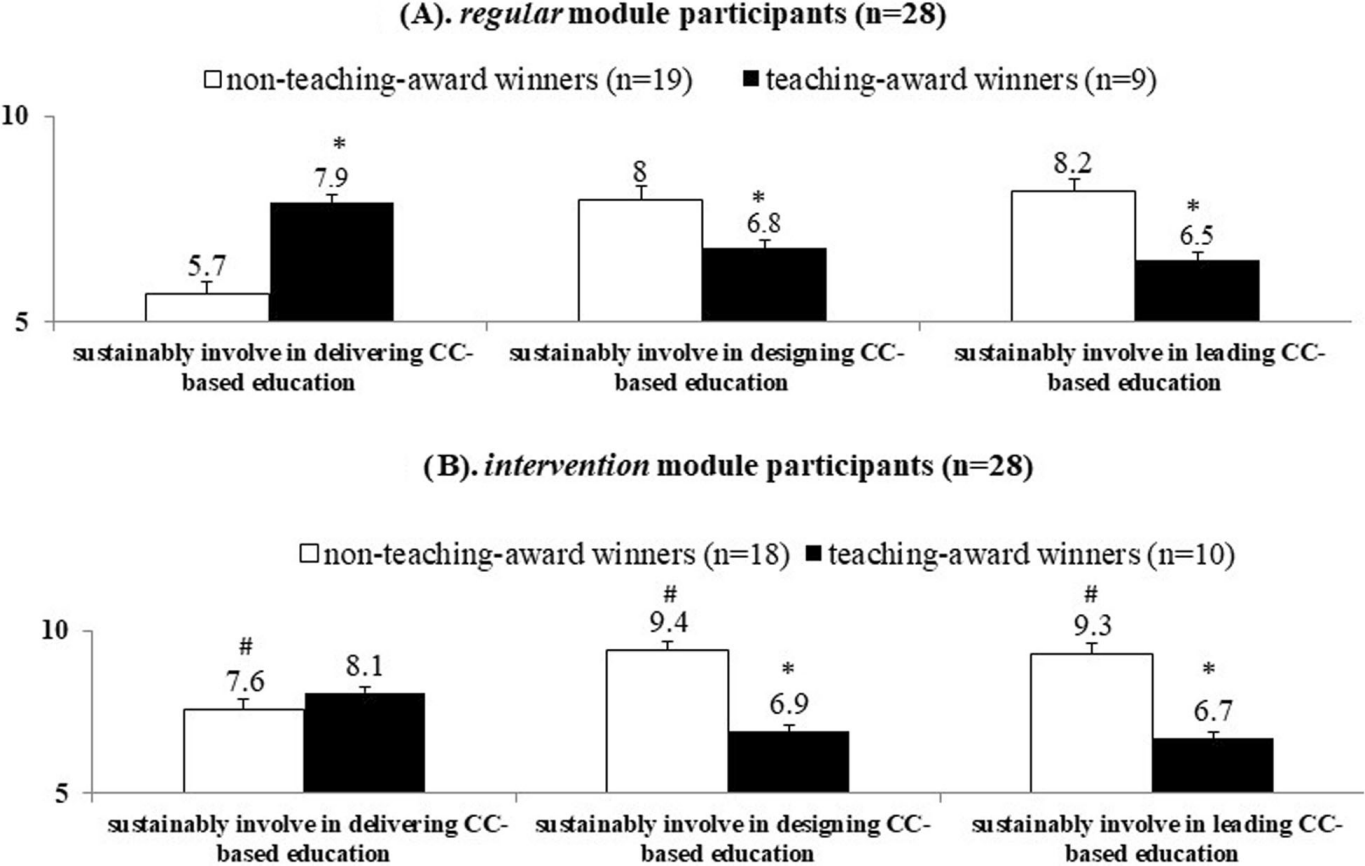
Comparison of the follow-up degree of sustained involvement in core competency (CC)-based courses between non-teaching award winners and teaching award winners participating in either (**a**) *regular* or (**b**) *intervention* FD modules; * *P* < 0.05 vs. data of non-teaching-award winners; ^#^*P* < 0.05 vs. data compared to the data of the non-teaching award winners in the *regular* group were analyzed

Among non-teaching award winners of both groups, the follow-up degree of sustained involvement in *designing and leading* CC-based teachings or assessments was higher than the degree of involvement in *delivering* CC-based course (Fig. 3a&b). Among non-teaching-award winners, the follow-up degree of sustained involvement in *delivering, designing and leading* CC-based teachings or assessments in the *intervention* group was significantly higher than *regular* group (Fig. 3a&b). These results indicate that the *intervention* module effectively motivated non-teaching award winners toward more sustained involvement in *delivering*, *designing* and *leading* of CC-based teachings or assessments.

## Discussion

Today, clinical instructors need to be multifaceted cope with rapid changes in the medical educational system [10–12, 25]. Our one-year longitudinal study reveals that the *intervention* FD module motivated new clinical instructors to incorporate the trained skills to fit roles of instructors and leaders in CC education [6, 26].

Through the initial peer-supported learning-based exposure of 40-h course across 3 months and continued follow-up, our participants developed enthusiasm for CC education by forming a learning community of individuals whose having similar educational interests [6, 16, 27–29]. Our study suggests that the leadership-enhanced *intervention* module is feasible and acceptable for long-term faculty development.

*Teaching clinics,* are a more ideal environment than *bedside* for CC-based teaching. In our study, the effectiveness of *intervention* FD module was confirmed by a higher self-reported degree of familiarity with the skills of *delivering* CC-based *teaching clinics* in the *intervention* group than in the *regular* group (Table 3). However, our *intervention* FD module did not appropriately increase participants’ familiarity with *delivering* CC-based itinerant and circuit *bedside teaching.* In general, it is more challenging to train to delivery CC-based *bedside teaching*, as it is a more complex clinical environment than a *teaching clinics*. In *bedside teaching*, faculties must alternate among the roles of doctor, instructor and leader [30–32]. Accordingly, in future versions of the *intervention* module, it will be necessary to increase the proportion of training on aspects of *delivering* skills of CC-based *bedside teaching*.

In our institution, teaching awards winners are selected online by junior medical trainees according to the annual performance of teachers. Consequently, teaching award winners are considered to be more high teaching performance teachers than non-teaching award winners. Besides encouraging high teaching-performance teachers, teaching awards provide opportunities for teachers and program directors to review their teaching and programs. Teaching awards also motivate low teaching-performance teachers to improve themselves by receiving more training. In general, educational leaders in CC education tend to be high teaching-performance teachers as opposed to low teaching-performance teachers. Baroffio et al. have suggested that the ideal leadership FD program will to help the teaching performance and leadership of low teaching-performance teachers [33]. Engagement and serial evaluations have been reported as successful strategies for improving the teaching performance of low teaching-performance faculty [34, 35]. Thus, our *intervention* FD module, which emphasized *hands-on* experiences, guided reflection and serial evaluations, successfully motivated non-teaching award winners (low teaching performance teacher) to evolve substantially as leaders in CC education.

The Kirkpatrick Model is a well-known model for evaluating the effects of FD programs. *Level 1 (reaction)* of the Kirkpatrick Model measures participants’ satisfaction; *level 2 (earning)* analyzes whether the FD increases participants’ knowledge or skills (participants’ familiarity with and confidence in CC education); *level 3 (behavior)* looks at whether participants utilize what they learn at work (participants’ sustained involvement in CC education), and *level 4 (results)* determines whether the FD had a positive impact on the organization.

### Strengths and limitations

The limitations of this study include the following. First, it did not assess outcomes at level 4 of the Kirkpatrick Model such as whether the *intervention* FD module improved clinical education or decreased medical errors [14, 15, 36–39]. Therefore, it will be necessary to assess such parameters in the future. On the other hand, self-reporting data is valuable for obtaining subjects’ perspectives and views, but it has potentially selection and recall bias [40]. To avoid such bias, in our study, the self-reported questionnaires were completed by voluntary participants immediately before and after the training and that can be avoided from. Usually, voluntary participants have stronger motivation than other participants which can result in a selection bias. Nevertheless, the similar questionnaires, follow-up duration and criteria selection for enrollment of participants between *regular* and *intervention* groups in our study can partially overcome the possible bias of self-reported data. A second limitation was the fact that the *intervention* FD group completed leadership training and some of the questions on the surveys were leadership-related. To have comparable results, both the *regular* and *intervention* FD groups used similar questionnaires for self-assessment in our study. Still, the multiple self-assessment time points in the current study indicated sustained effects of the *intervention* FD module on leadership training of clinical instructors in *delivering*, designing and *leading* CC-based teaching and assessment.

## Conclusions

Faced with continuous changes in medical education and practice, the new generation of medical educators is required to be not only clinical instructors but also educational leaders. Despite some limitations, the present study confirmed the effectiveness of leadership-enhanced *intervention* FD modules to motivate new clinical instructors to become substantially involve as leaders in ACGME CC-based education.

## Data Availability

The datasets used and/or analysed during the current study available from the corresponding author on reasonable request.

## Abbreviations

CC: Core competence
ACGME: Accreditation Council for Graduate Medical Education
CBD: Case-based discussion
mini-CEX: Mini-clinical evaluation exercise
OSCE: Objective structured clinical examination
